# Supplementary material for: Factors Predicting 30-Day Grade IIIa–V Clavien–Dindo Classification Complications and Delayed Chemotherapy Initiation after Cytoreductive Surgery for Advanced-Stage Ovarian Cancer: A Prospective Cohort Study
Source: Cancers (Basel). 2022 Aug 29;14(17):4181. doi: 10.3390/cancers14174181 (PMC9454550; doi:10.3390/cancers14174181)
Supplement: Supplementary file 1 [file cancers-14-04181-s001.zip › cancers-1816681-supplementary.pdf]

cancers

Supplementary table

Table S1. Enhanced recovery after surgery (ERAS) protocol

Table S2. Clavien-Dindo Classification

Table S3. Type and frequency of surgical procedures (*n*=300)

Table S4. Type and frequency of intra-operative complications

TableS5. Factors related to Clavien-Dindo classification ≥IIIa complications in ICS patients

TableS6. Factors related to time to initiating chemotherapy >42 days in ICS patients

Supplementary

Table S1. Enhanced recovery after surgery (ERAS) protocol

Time	ERAS Society recommendation for gynecologic surgery
Preoperative period<br><br>Outpatient clinic	- Preoperative patients' education and risk stratification<br><br>- Smoking and alcohol cessation
Admission	- Avoid mechanical bowel preparation<br><br>- Fasting up to 6 hours before anesthetic induction<br><br>- Oral carbohydrate solution loading up to 2 hours before anesthetic induction<br><br>- Avoid routine sedative use<br><br>- Thromboembolism prophylaxis
Day of surgery	- Insertion of Foley catheter<br><br>- Surgical antibiotic prophylaxis<br><br>- Thromboembolism prophylaxis<br><br>- Short-active anesthetic<br><br>- Goal-directed/Balanced fluid therapy<br><br>- Prevention of hypothermia<br><br>- Multi-modal analgesia and antiemetics<br><br>- Avoid routine drain placement<br><br>- Continue and encourage ambulation<br><br>- Early oral feeding adapted to patients' habits and tolerances within 24 hours
Post operation period	- Thromboembolism prophylaxis<br><br>- Remove of Foley catheter as early as possible

	- Remove drain as early as possible<br>- Continue and encourage ambulation<br>- Multimodal analgesia (opioid sparing pain therapy)

Table S2. Clavien-Dindo Classification

Clavien-Dindo grades	Definition
**Grade I**	Any deviation from the normal postoperative course without the need for pharmacological treatment or surgical, endoscopic and radiological interventions<br>Allowed therapeutic regimens are: drugs as antiemetics, antipyretics, analgesics, diuretics and electrolytes and physiotherapy.<br>This grade also includes wound infections opened at the bedside.
**Grade II**	Requiring pharmacological treatment with drugs other than such allowed for grade I complications.<br>Blood transfusions and total parenteral nutrition are also included.
**Grade III**	Requiring surgical, endoscopic or radiological intervention
**Grade IIIa**	Intervention not under general anesthesia
**Grade IIIb**	Intervention under general anesthesia
**Grade IV**	Life-threatening complication (including CNS complications) * requiring IC/ICU-management
**Grade IVa**	single organ dysfunction (including dialysis)
**Grade IVb**	Multiorgan dysfunction
**Grade V**	Death of a patient

*Brain hemorrhage, ischemic stroke, subarachnoid bleeding, but excluding transient ischemic attack, IC: intermediate care, ICU: intensive care unit

Table S3. Type and frequency of surgical procedures (*n*=300)

Procedure	N (%)
**Upper abdominal surgery**	
Diaphragmatic peritonectomy	36 (12.0)
Partial hepatic resection	39 (13.0)
Partial splenectomy	11 (37.0)
Total splenectomy	11 (37.0)
**Bowel surgery**	
Resection of tumor spot at small bowel	88 (67.3)
Small bowel resection	10 (3.3)
Resection of tumor spot at rectosigmoid	107 (35.7)
Rectosigmoid resection	47 (15.7)
Resection of tumor spot at cecum	70 (23.3)
Ileocecal resection	12 (4.0)
Colostomy	29 (9.7)
Appendectomy	156 (52.0)
Resection of tumor at mesentery	156 (52.0)
**Bladder surgery**	138 1(46)
Resection of tumor at bladder	137 (45.7)
Bladder resection	1 (0.3)
**Lymph node surgery**	
Pelvic lymphadenectomy	36 (12)
Paraaortic lymphadenectomy	21 (7.0)

One patient can endure more than one procedure

Table S4. Type and frequency of intra-operative complications (*n*=300)

**Type**	**N (%)**
Urinary tract injury	12 (4.0)
Bowel injury	21 (7.0)
Pneumothorax	8 (2.7)
Upper abdominal visceral injury*	10 (3.3)
Blood loss >1L	125 (41.6)

*Upper abdominal visceral injury: pancreas, stomach, liver or spleen

Table S5. Factors related to Clavien-Dindo classification ≥IIIa complications in ICS patients

Variables	Univariable analysis unadjusted OR (95%CI)	*P* value	Multivariable analysis Adjusted OR (95%CI)	*P* value
**Pre-operative factor**				
Age (per 5-year increase)	1.18 (0.97-1.42)	0.09	1.20 (0.96-1.50)	0.12
BMI (per kg/m^2 increase)	0.99 (0.93-1.07)	0.92		
WHO performance status ≥2	0.77 (0.28-2.12)	0.62		
Daily smoker	1.85 (0.68-5.06)	0.23		
Diabetes mellitus	1.51 (0.84-2.72)	0.17		
Hypertension	1.63 (0.82-3.23)	0.16		
**Cardiovascular disease***	**3.14 (1.55-6.38)**	**0.002**	**4.01 (1.66-9.68)**	**0.002**
**Intra-operative procedure**				
Pelvic peritonectomy	1.69 (0.88-3.27)	0.12		
Bladder surgery	1.62 (0.85-3.07)	0.14		
Small bowel surgery	1.06 (0.60-1.88)	0.85		
Colon surgery	1.95 (0.99-3.83)	0.05	1.23 (0.51-2.97)	0.65
Appendectomy	1.59 (0.80-3.16)	0.18		
Mesenteric resection	1.69 (0.88-3.27)	0.12		
Partial hepatectomy	0.99 (0.38-2.58)	0.99		
Splenectomy	1.43 (0.49-4.15)	0.51		
Pelvic lymph node resection	1.62 (0.67-3.91)	0.28		
Paraaortic lymph node resection	1.43 (0.44-4.64)	0.56		
**Diaphragmatic surgery**	**3.81 (1.96-7.43)**	**<0.001**	**3.90 (1.74-8.70)**	**0.001**
Colostomy	2.86 (1.17-7.01)	0.022	2.42 (0.77-7.63)	0.13
HIPEC procedure	1.07 (0.50-2.26)	0.87		
Debulking with PlasmaJet	0.62 (0.33-1.17)	0.14	1.08 (0.98-1.20)	0.11
Operative time (per 30-min increase)	1.12 (1.03-1.21)	0.005		
**Intra-operative injury**				
**Urinary tract injury**	**4.69 (1.30-16.93)**	**0.018**	**6.90 (1.50-31.69)**	**0.013**
Bowel injury	2.54 (0.89-7.26)	0.08	0.83 (0.21-3.36)	0.80
**Upper abdominal visceral injury****	**4.61 (1.11-19.14)**	**0.04**	**9.97 (1.53-65.08)**	**0.016**
Pneumothorax	3.38 (0.73-15.62)	0.12		
Blood loss >1L	1.92 (0.99-3.68)	0.05	1.15 (0.49-2.69)	0.76
**Post-operative factor**				
Complete cytoreduction	1.08 (0.48-2.40)	0.86		
FIGO stage				
Stage IIIB (reference)				

Stage IIIC	1.56 (0.33-7.35)	0.57
Stage IV	1.135 (0.23-5.64)	0.88

BMI: Body mass index, WHO: world health organization, HIPEC: hyperthermic intraperitoneal chemotherapy FIGO: International federation of obstetrics and gynecology, * cardiovascular disease was defined as any of the following disease: myocardial infarction, stroke, peripheral vascular disease, **Upper abdominal visceral injury: pancreas, stomach, liver or spleen. A bold font denotes factors that are significant in both a univariate and multivariate model.

TableS6. Factors related to time to initiating chemotherapy >42 days in ICS patients

Variables	Univariable analysis unadjusted OR (95%CI)	*P* value	Multivariable analysis adjusted OR (95%CI)	*P* value
**Pre-operative factor**				
Age (per 5-year increase)	0.96 (0.81-1.13)	0.59		
BMI (per kg/m2 increase)	1.02 (0.96-1.08)	0.47		
**WHO performance status ≥2**	**1.59 (0.69-3.64)**	**0.27**		
Daily smoker	2.24 (0.86-5.85)	0.10		
Presence of comorbidity*	1.52 (0.80-2.88)	0.20		
**Intra-operative factor**				
Extensive surgery**	0.98 (0.52-1.85)	0.95		
Debulking with PlasmaJet	0.81 (0.45-1.44)	0.81		
HIPEC procedure	2.17 (1.12-4.18)	0.02		
Operative time (per 30-min increase)	1.07 (0.99-1.15)	0.09		
**Intra-operative injury**				
Urinary tract injury	3.80 (0.99-14.62)	0.05		
**Bowel injury**	**4.13 (1.47-11.63)**	**0.007**		
**Upper abdominal visceral injury*** **	**7.68 (1.45-40.68)**	**0.017**		
Pneumothorax	6.04 (1.08-33.83)	0.041		
Blood loss >1L	1.06 (0.57-1.95)	0.86		
**Post-operative factor**				
Complete cytoreduction	0.77 (0.37-1.59)	0.48		
FIGO stage				
Stage IIIB (reference)				
Stage IIIC	1.23 (0.32-4.77)	0.77		
Stage IV	0.79 (0.19-3.22)	0.74		
**Post-operative complications CDC ≥IIIa**	**3.45 (1.74-6.85)**	**<0.001**		

BMI: Body mass index, WHO: world health organization, FIGO: International federation of obstetrics and gynecology, HIPEC: hyperthermic intraperitoneal chemotherapy, CDC: Clavien-Dindo classification,*Presence of comorbidity: ≥1 of the following comorbidity: diabetes mellitus, hypertension, cardiac disease,**Extensive surgery was defined as any of the following procedures: peritonectomy, diaphragmatic peritonectomy, resection of subcapsular liver metastases, splenectomy, bowel resection or resection of extra-abdominal metastatic sites. ***Upper abdominal visceral injury: any injury of pancreas, stomach, liver or spleen. A bold font denotes factors that are significant in both a univariate and multivariate model.
